# Combined inhibition of aurora kinases and Bcl-xL induces apoptosis through select BH3-only proteins

**DOI:** 10.1016/j.jbc.2023.102875

**Published:** 2023-01-06

**Authors:** Jian Li, Cheng-Hsun Chen, Katelyn L. O’Neill, Valerie J. Fousek-Schuller, Adrian R. Black, Jennifer D. Black, Jingjing Zhang, Xu Luo

**Affiliations:** 1 Eppley Institute for Research in Cancer and Allied Diseases, Fred & Pamela Buffett Cancer Center, Omaha, Nebraska, USA; 2 Department of Pathology & Microbiology, University of Nebraska Medical Center, Omaha, Nebraska, USA

**Keywords:** aurora kinases, aurora kinase inhibitor, Bcl-2 family, apoptosis, BH3-mimetics, caspases, AURK, aurora kinase, BH, Bcl-2 homology, DKO, double KO, DMSO, dimethyl sulfoxide, Ni–NTA, nickel–nitrilotriacetic acid, PI, propidium iodide, tBid, truncated Bid

## Abstract

Aurora kinases (AURKs) are mitotic kinases important for regulating cell cycle progression. Small-molecule inhibitors of AURK have shown promising antitumor effects in multiple cancers; however, the utility of these inhibitors as inducers of cancer cell death has thus far been limited. Here, we examined the role of the Bcl-2 family proteins in AURK inhibition–induced apoptosis in colon cancer cells. We found that alisertib and danusertib, two small-molecule inhibitors of AURK, are inefficient inducers of apoptosis in HCT116 and DLD-1 colon cancer cells, the survival of which requires at least one of the two antiapoptotic Bcl-2 family proteins, Bcl-xL and Mcl-1. We further identified Bcl-xL as a major suppressor of alisertib- or danusertib-induced apoptosis in HCT116 cells. We demonstrate that combination of a Bcl-2 homology (BH)3-mimetic inhibitor (ABT-737), a selective inhibitor of Bcl-xL, Bcl-2, and Bcl-w, with alisertib or danusertib potently induces apoptosis through the Bcl-2 family effector protein Bax. In addition, we identified Bid, Puma, and Noxa, three BH3-only proteins of the Bcl-2 family, as mediators of alisertib–ABT-737-induced apoptosis. We show while Noxa promotes apoptosis by constitutively sequestering Mcl-1, Puma becomes associated with Mcl-1 upon alisertib treatment. On the other hand, we found that alisertib treatment causes activation of caspase-2, which promotes apoptosis by cleaving Bid into truncated Bid, a suppressor of both Bcl-xL and Mcl-1. Together, these results define the Bcl-2 protein network critically involved in AURK inhibitor–induced apoptosis and suggest that BH3-mimetics targeting Bcl-xL may help overcome resistance to AURK inhibitors in cancer cells.

Aurora kinases (AURKs) are a family of serine/threonine kinases that regulate cell cycle progression through their activities in mitosis. Three family members, AURK A, B, and C, sharing a high degree of sequence homology, have been identified in human and shown to play key roles in chromosomal arrangement and mitotic spindle formation during cell division (1, 2). AURK A is involved in mitosis entry, centrosome maturation, and spindle formation (3). In addition to its mitotic functions, aurora A has also been shown to be involved in DNA damage/repair, DNA replication, regulation of p53/p73, and so on (4). AURK B is a component of the chromosomal passenger protein complex known to be involved in chromosomal condensation, chromosome biorientation, and cytokinesis (2). Aurora C appears to have similar functions as AURK B and is primarily expressed in testis (5).

Both aurora A and B are frequently overexpressed (gene amplification, inhibition of degradation, etc.) in various cancers, including breast, ovary, colon, pancreas, prostate, etc., and have been shown to promote genome instability, aneuploidy, oncogenesis, and drug resistance (6, 7). Inactivation of AURK A by genetic or pharmacological approaches has been shown to prevent the progression of various tumors and suppress cancer cell proliferation by causing mitotic spindle pole fragmentation, cytotoxicity, and DNA damage–induced senescence (8, 9, 10, 11). Numerous small-molecule inhibitors against AURK A and/or B and C have been developed and shown to have antitumor effects *in vitro* and *in vivo* (12). Many of these inhibitors have entered clinical trials as single agents or as combinatorial partners with other agents, and the most advanced AURK inhibitor in clinical trials is MLN8237 (alisertib), an ATP-competitive and highly selective inhibitor of AURK A with an IC_50_ of 1 nM (7). Preclinical studies have found that alisertib has excellent antitumor activities and inhibits cell proliferation mainly by inducing cell senescence (13). Importantly, alisertib has been shown to cooperate with many other compounds to inhibit tumor growth (13). Clinical trials for alisertib ranging from phase I to phase III have been conducted against different solid or hematological tumors and have demonstrated promising effects (14). Danusertib is an ATP-competitive pan-AURK inhibitor that exerts strong activity against aurora A, B, and C (12). In preclinical studies, danusertib exhibited antiproliferative activities that include cell cycle arrest, apoptosis, and autophagy (7). Several phase I/II clinical trials have been conducted with encouraging results (12).

Inhibition of aurora A or B is known to induce defects in chromosomal segregation, mitotic arrest, and mitotic catastrophe, which is a process in which malfunction of the mitosis machinery causes an array of chromosomal defects that eventually lead to senescence, polyploidy, and cell death (1, 15, 16). Several cell cycle and cell death regulators, including AURKs, p21, p53, caspase-2, Bid, etc., have been shown to be involved in mitotic catastrophe and eventual cell death (15). However, the communication between the mitotic and the apoptosis machineries following the onset of mitotic catastrophe has not been fully elucidated.

The Bcl-2 family proteins are major regulators and effectors of mitochondria-dependent apoptosis. There are 17 to 18 commonly accepted Bcl-2 family members, and all of them contain at least one of the four Bcl-2 homology (BH) domains, termed BH1 through BH4 (17). While the antiapoptotic members, including Bcl-2, Bcl-xL, Mcl-1, Bcl-w, and A1, are prosurvival, the effector proteins (Bax, Bak, and possibly Bok) and BH3-only proteins (Bid, Bim, Puma, Hrk, Bmf, Noxa, Bik, and Bad) are proapoptotic (18). It is currently understood that upon receiving apoptotic signals, cells activate the BH3-only proteins, generally considered the sentinels of cellular stress (19, 20), and the activated BH3-only protein in turn target and suppress the antiapoptotic Bcl-2 family proteins (21), which normally suppress the spontaneous activation of Bax and Bak (22, 23). The BH3-only–mediated inactivation of these antiapoptotic proteins, in most cases, Bcl-xL and Mcl-1, leads to the unimpeded, membrane-mediated, spontaneous activation of Bax, Bak (22, 23, 24, 25). Several of the BH3-only proteins have also been reported to be able to directly bind to and activate Bax/Bak (26, 27, 28). Once activated, Bax/Bak form pores in the mitochondrial outer membrane that allow the release of apoptogenic proteins, such as cytochrome *c*, which triggers formation of the apoptosome, leading to activation of the effector caspases and apoptosis (29, 30).

How the apoptosis machinery senses defects in the mitotic machinery following inhibition of the AURKs and how mitotic catastrophe induces apoptosis have remained sketchy. In addition, what can be used in combination with these AURK inhibitors to enhance their capabilities of cell elimination? In this study, the ability of two AURK inhibitors, alisertib and danusertib, to induce apoptosis in colon cancer cells is investigated. We identify the Bcl-2 family proteins critically involved in the apoptotic responses following treatment by these inhibitors and suggest the combination of a BH3-mimetic and the AURK inhibitors as a combinatorial approach for the induction of apoptosis in colon cancer cells.

## Results

### Alisertib and danusertib are inefficient inducers of apoptosis in colon cancer cells but potently induce apoptosis when combined with a BH3-mimetic

**Figure 1:**
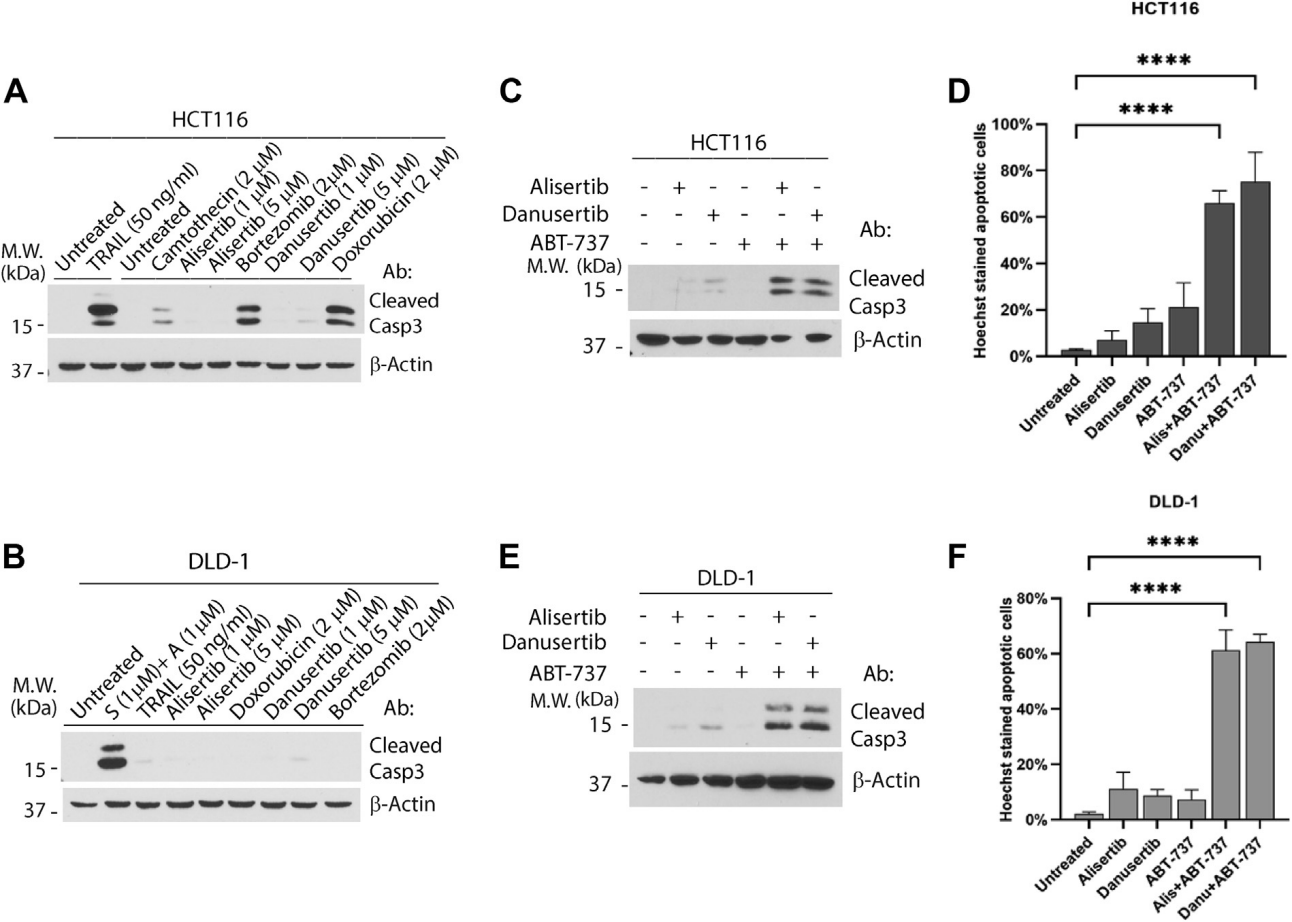
**Alisertib and danusertib are inefficient inducers of apoptosis in colon cancer cells but potently induce apoptosis when combined with a BH3-mimetic.***A*, HCT116 cells were treated with TRAIL for 6 h or with camptothecin, alisertib, bortezomib, and danusertib, or doxorubicin at the indicated concentrations for 24 h. Cells were harvested to generate whole-cell lysates for Western blot analysis. *B*, DLD-1 cells were treated with S (S63845) + A (ABT-737) for 6 h or with TRAIL, alisertib, doxorubicin, danusertib, or bortezomib at the indicated concentrations for 24 h. Cells were harvested, and the whole-cell lysate was for used for Western blot analysis. *C*, HCT116 cells were treated with alisertib (1 μM), danusertib (1 μM) for 24 h, and with ABT-737 (1 μM) for 6 h. Cells were harvested, and the whole-cell lysate was used for Western blot analysis. *D*, HCT116 cells were treated as described in (*C*) and were stained by Hoechst dye for 30 min at the end of the treatments. Cells underwent apoptosis were detected as those that show nuclear condensation after Hoechst staining. Apoptosis was quantified by counting the percentage of apoptotic cells among total number of cells in a picture. Mean and SD values were shown on the graph, n = 3. *E*, DLD-1 cells were treated as described in (*C*). Cells were harvested, and the whole-cell lysate was used for Western blot analysis. *F*, DLD-1 cells were treated as described in (*C*) and stained by Hoechst dye for 30 min at the end of the treatments. Cells underwent apoptosis were quantified. Mean and SD values were shown on the graph, n = 3. Representatives of at least three independent experiments were shown for Western blots. One-way ANOVA was used to test the significance, *p* < 0.0001 was defined as significant and indicated with ∗∗∗∗.

AURKs are required for proper mitosis, and their inhibition is known to cause mitotic catastrophe and eventually senescence and/or apoptosis (3). We examined the apoptotic activities of the two aurora A kinase inhibitors, alisertib and danusertib, in two colon cancer cell lines HCT116 and DLD-1. Unlike the common apoptosis inducers TRAIL, bortezomib, and camptothecin, alisertib and danusertib displayed very weak apoptotic activities against HCT116 and DLD-1 cells after 24 h, with minimal caspase-3 cleavage detected (Fig. 1, *A* and *B*). We hypothesize that this insensitivity is due to a failure of these cells to engage the mitochondria-dependent apoptosis pathway in response to these treatments. We therefore tested whether ABT-737, a BH3-mimetic known to selectively inhibit Bcl-xL, Bcl-2, and Bcl-w (31), can assist alisertib or danusertib in killing these cells. Although the addition of ABT-737 alone has little apoptotic activity in HCT116 cells and modest activity in DLD-1 cells, the combination between alisertib or danusertib with ABT-737 robustly induces apoptosis in both HCT116 and DLD-1 cells (Fig. 1, *C*–*F*). Of note, similar results were obtained when this experiment was carried out with Annexin V staining (Fig. S1). Together, these results strongly suggest that Bcl-xL, Bcl-2, or Bcl-w, either alone or in combination, suppresses alisertib/danusertib-induced apoptosis.

### Bcl-xL is a major suppressor of alisertib/danusertib-induced apoptosis in HCT116 cells

**Figure 2:**
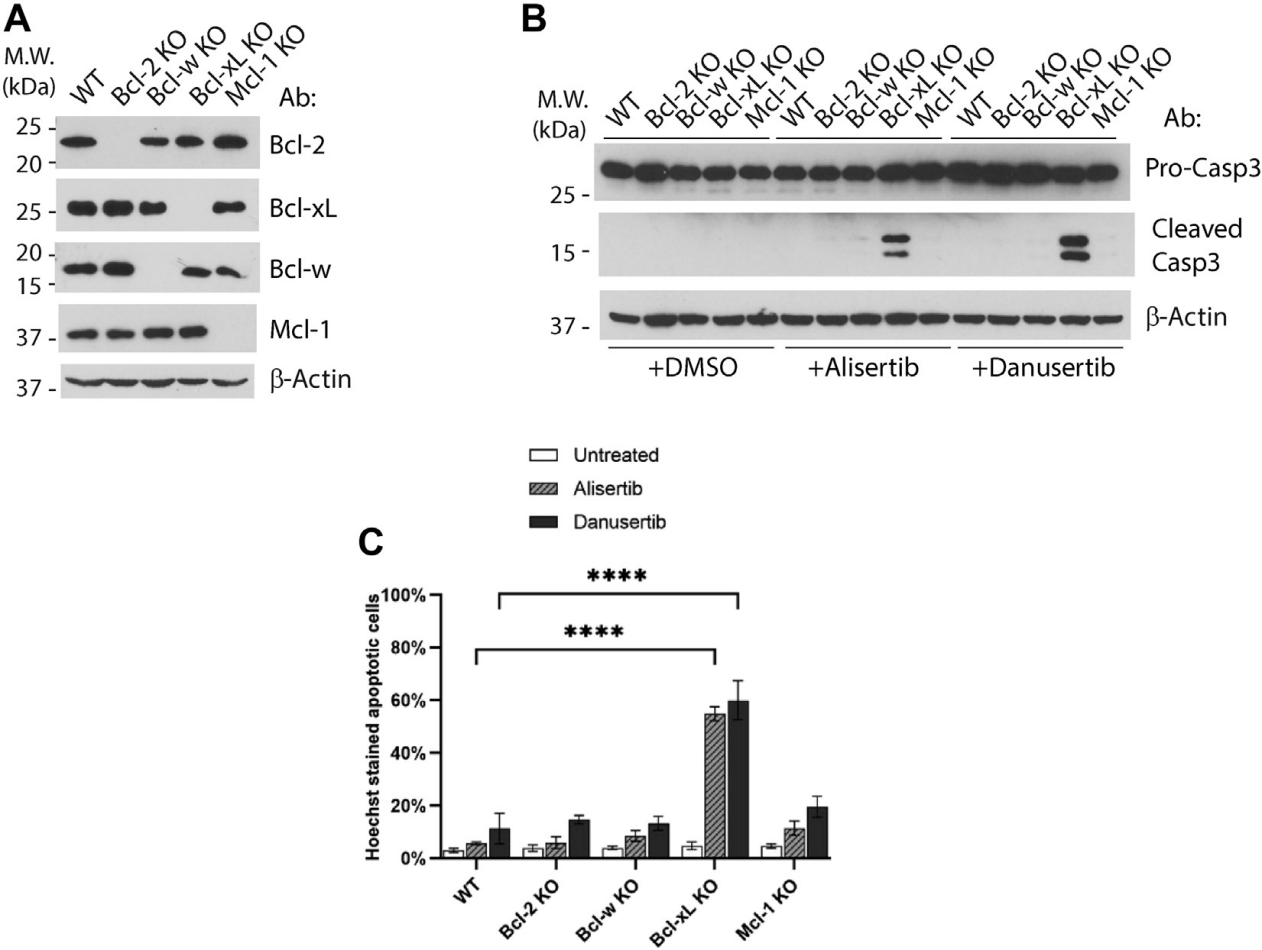
**Bcl-xL is a major suppressor of alisertib–danusertib-induced apoptosis in HCT-116 cells.***A*, single clones of Bcl-2 KO, Bcl-w KO, Bcl-xL KO, and Mcl-1 KO cells were generated from HCT116 cells by CRISPR–Cas9. Cells were harvested, and the whole-cell lysate was used to examine KO by Western blot. *B*, HCT116 WT and single KO cells established in (*A*) were treated with alisertib (1 μM) and danusertib (1 μM) for 24 h, and cells were harvested and the whole-cell lysate was used for Western blot analysis. *C*, same cell lines and treatments as described in (*B*) were subjected to Hoechst staining for quantification of apoptotic cells. Mean and SD values were shown on the graph, n = 3. Representatives of at least three independent experiments were shown for Western blots. Two-way ANOVA was used to test the significance, *p* < 0.0001 was defined as significant and indicated with ∗∗∗∗.

A loss-of-function approach was used to identify the antiapoptotic Bcl-2 family proteins responsible for suppressing alisertib/danusertib-induced apoptosis in HCT116 cells. Single clones of HCT116 cells deficient for Bcl-xL, Bcl-2, Bcl-w, or Mcl-1 were generated through CRISPR–Cas9 (Fig. 2*A*) and tested for their response to alisertib or danusertib. While Bcl-2 KO, Bcl-w KO, or Mcl-1 KO cells, similar to the wildtype HCT116 cells, had minimal responses, Bcl-xL KO cells displayed robust apoptotic response to the treatment of alisertib or danusertib (Fig. 2, *B* and *C*). This result strongly suggests that Bcl-xL, but not Bcl-2, Bcl-w, or Mcl-1, plays a major role in suppressing A/D-induced apoptosis in HCT116 cells and is the main reason for the insensitivity of these cells to these drugs. Equally important, these results indicate that Bcl-xL, but not the functionally similar proteins Bcl-2 or Bcl-w, is the only relevant target of ABT-737 in these cells.

To further explore the prominent role of Bcl-xL in this pathway, we assessed the relative amounts of endogenous Bcl-xL and Bcl-2 by comparing them with their respective GFP-fusion proteins expressed in HCT116 cells. The amount of endogenous Bcl-xL is estimated to be fivefold to eightfold of Bcl-2, suggesting that one of the reasons for Bcl-xL to play a major role in this pathway is that it is expressed at a higher level than Bcl-2 (Fig. S2). However, it remains possible that at endogenous levels, Bcl-xL has a stronger antiapoptotic activity than Bcl-2.

### Combination of alisertib or danusertib with ABT-737 induces apoptosis through Bax but not Bak

**Figure 3:**
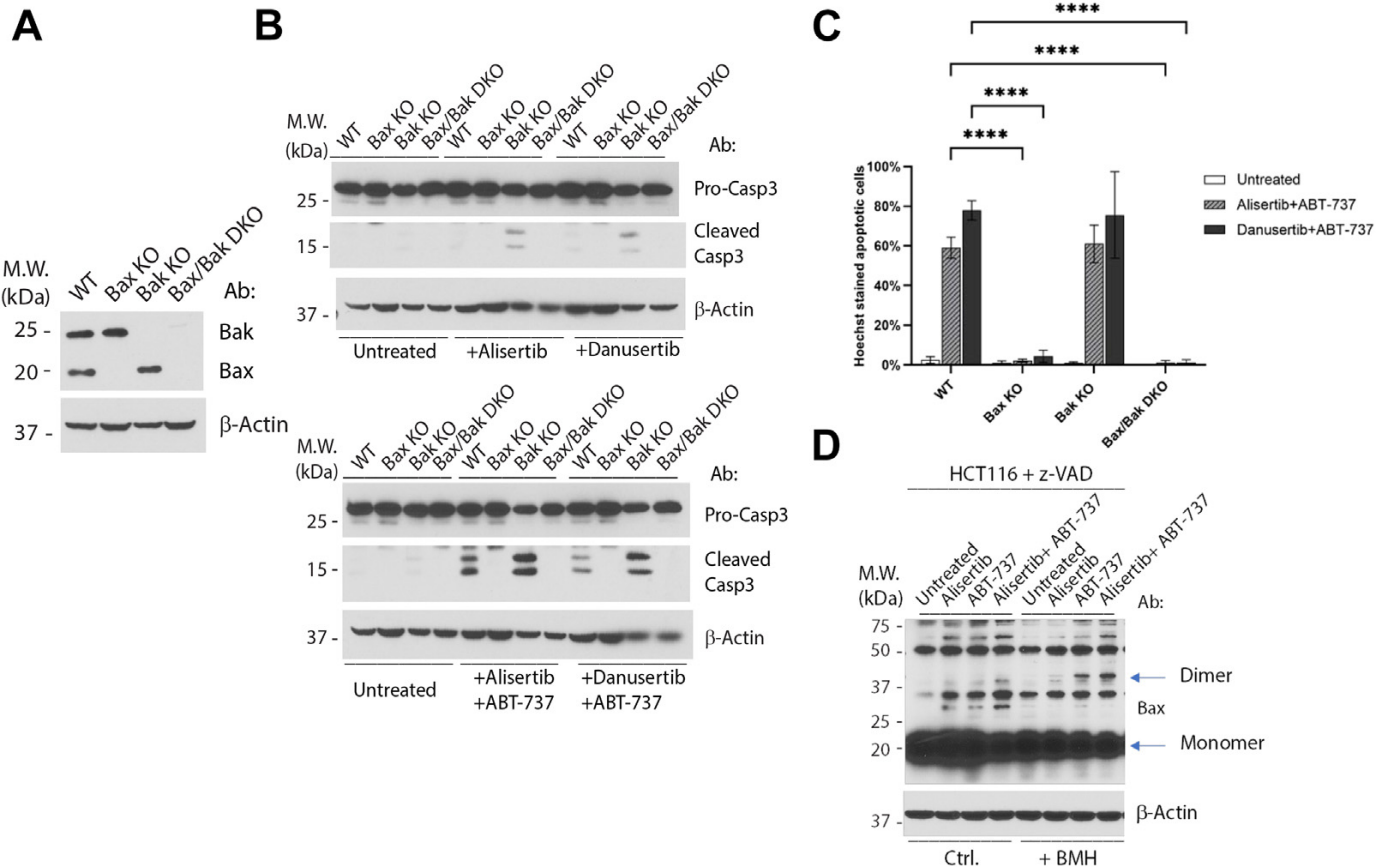
**Combination of alisertib–danusertib and ABT-737 induces apoptosis through Bax but not Bak.***A*, Bax KO, Bak KO, and Bax/Bak double KO (DKO) were generated from HCT116 cells by CRISPR–Cas9. Cells were harvested, and the whole-cell lysate was used to examine KO by Western blot. *B*, WT and KO cells established in (*A*) were treated with alisertib–danusertib (1 μM) for 24 h with or without ABT-737 (1 μM) for 6 h. Cells were harvested, and the whole-cell lysate was used for Western blot analysis. *C*, same cells and treatments as described in (*B*), and cells were subjected to Hoechst staining for quantification of apoptotic cells. Mean and SD values were shown on the graph, n = 3. Representatives of at least three independent experiments were shown for Western blots. Two-way ANOVA was used to test the significance, *p* < 0.0001 was defined as significant and indicated with ∗∗∗∗. *D*, HCT116 cells were treated with the listed reagents in the presence of z-VAD-fmk (50 μM). Alisertib and ABT-737 were both added at 1 μM. Cell lysates with incubated with 1,6-bismaleimidohexane (BMH; 200 nM) and analyzed by Western blot as described in the Experimental procedures section.

Next, we investigated whether apoptosis induced by the combinatorial treatment of alisertib and ABT-737 is through the intrinsic (mitochondria-dependent) pathway. As Bax and Bak are effectors of the mitochondria-dependent pathway (32), we tested the requirement of Bax and/or Bak in apoptosis induced by the combination of alisertib/danusertib and ABT-737. Single clones of Bax KO and Bak KO HCT116 cells were generated through CRISPR–Cas9 (Fig. 3*A*). Along with Bax/Bak double KO (DKO) cells generated from our previous publications (23), we tested the HCT116 wildtype, Bax KO, and Bak KO cells for their response to the combination treatment by alisertib/danusertib and ABT-737. While wildtype and Bak KO cells are sensitive, both Bax KO and Bax/Bak DKO cells are highly refractory to the combined treatment (Fig. 3, *B* and *C*), indicating that the combinatorial treatment induces apoptosis through a Bax-dependent mitochondrial pathway.

To further examine whether the mitochondrial pathway is engaged following the combinatorial treatment, we examined the dimerization of Bax, an indicator of Bax activation and the engagement of the mitochondrial pathway of apoptosis (25). Through the use of the crosslinker 1,6-bismaleimidohexane, we found that while alisertib alone is unable to induce Bax dimerization, and yet the alisertib–ABT-737 combination caused dimerization of Bax (Fig. 3*D*). Of note, ABT-737 alone also induced a modest level of Bax dimerization. Nonetheless, these results indicate that the mitochondrial apoptosis pathway is engaged following the combinatorial treatment of alisertib and ABT-737.

### Bid, Puma, and Noxa are necessary for alisertib–ABT-737-induced apoptosis

**Figure 4:**
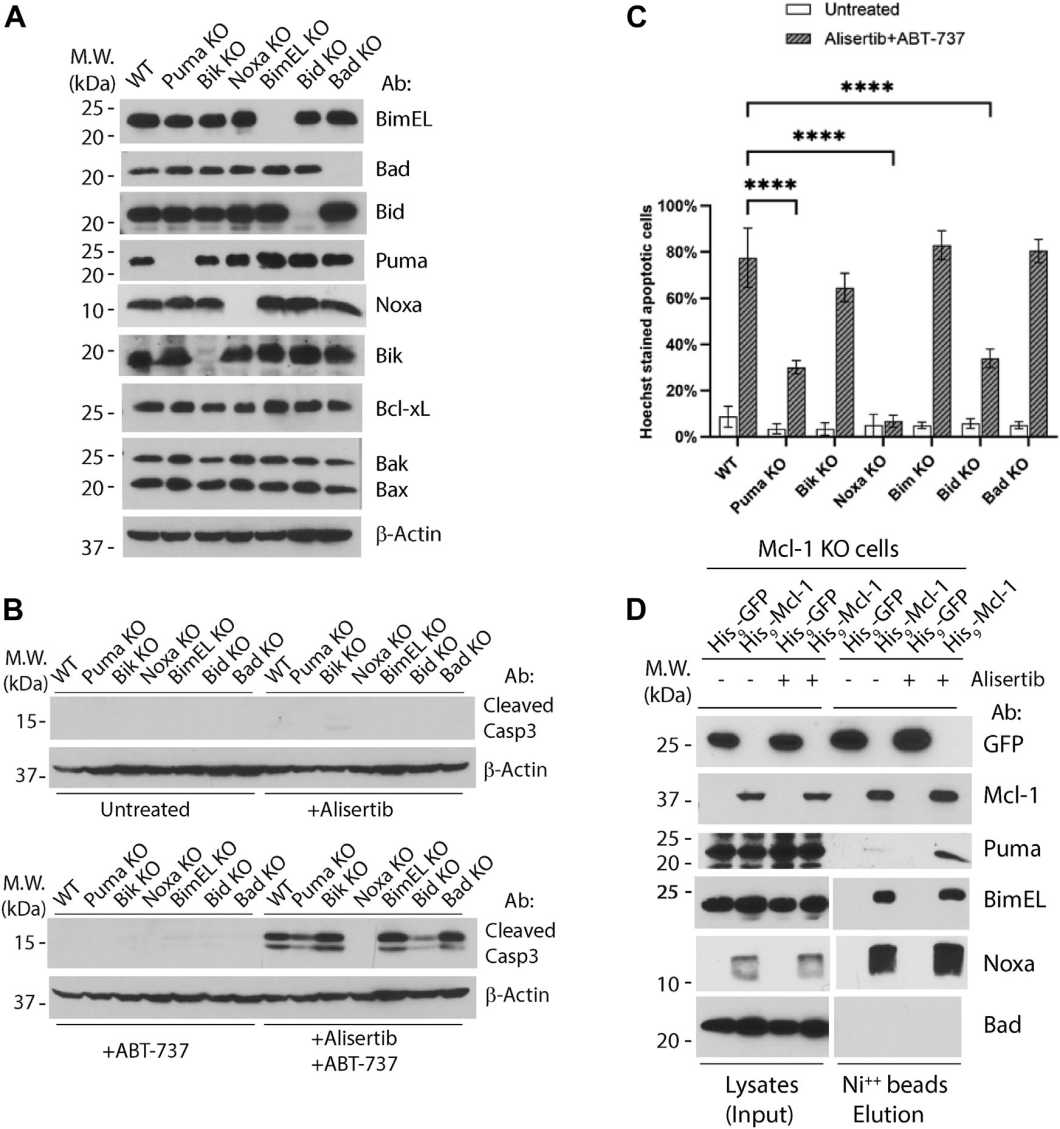
**Bid, Puma, and Noxa are necessary for alisertib–ABT-737-induced apoptosis.***A*, Puma KO, Bik KO, Noxa KO, Bid KO, and Bad KO were generated from HCT116 cells by CRISPR–Cas9; BimEL KO was established from HCT116 cells by TALEN. The whole-cell lysates were used to examine KO by Western blot. *B*, WT and KO cells established in (*A*) were treated with alisertib (1 μM) for 24 h with or without ABT-737 (1 μM) for 6 h. Cells were harvested, and the whole-cell lysate was used for Western blot analysis. *C*, same cells and treatments as described in (*B*) were subjected to Hoechst staining for quantification of apoptotic cells. Mean and SD values were shown on the graph, n = 2. *D*, expression of 9His-GFP and 9His-Mcl-1 in Mcl-1 KO cells was by retroviral infection, and cells were treated with alisertib (1 μM) for 24 h. Cells were harvested, and the whole-cell lysate was the input of nickel beads pull-down assay, 9His-GFP and 9His-Mcl-1 bound to nickel beads were eluted by 100 mM imidazole in EBC buffer, and the whole lysate and eluted protein were used for Western blot analysis. Representatives of at least three independent experiments were shown for Western blots. Two-way ANOVA was used to test the significance, *p* < 0.0001 was defined as significant and indicated with ∗∗∗∗.

To identify the upstream factors responsible for the activation of Bax following the combinatorial treatment, we used a loss-of-function approach to screen the BH3-only proteins for their role in this pathway. Through CRISPR–Cas9, we generated single clones of HCT116 cells deficient for Bid, Bim, Puma, Bik, Bad, or Noxa (Fig. 4*A*). When treated with the alisertib–ABT-737 combination, Bim KO, Bad KO, and Bik KO cells responded similarly to wildtype cells, but Bid KO, Puma KO, and Noxa KO cells displayed significant blockade (Fig. 4, *B* and *C*), indicating the requirement of Bid, Puma, and Noxa in this apoptotic pathway.

To investigate the mechanism of how Bid, Puma, and Noxa participate in alisertib–ABT-737-induced apoptosis, it is necessary to examine the binding of these proteins to the two antiapoptotic Bcl-2 proteins, Bcl-xL and Mcl-1, whose simultaneous inhibition is known to be necessary and sufficient for apoptosis induction (23, 33, 34, 35). However, as ABT-737 inhibits Bcl-xL (31), suppression of Mcl-1 is presumably the main determinant for apoptosis response to the alisertib–ABT-737 combination. We therefore examined the binding of these proteins to Mcl-1 before and after alisertib treatment. We first generated HCT116 Mcl-1 KO cells stably expressing polyhistidine-tagged Mcl-1 through retroviral expression. After treatment with alisertib, cell lysates were generated and subjected to nickel beads pull-down, and the proteins associated with poly-His-tagged-Mcl-1 are examined by Western blot. Bad and Bid did not bind to Mcl-1, whereas Noxa, BimEL, and Puma bind to Mcl-1. Although Noxa and BimEL displayed a constitutive binding to Mcl-1, Puma displayed an induced binding following alisertib treatment (Fig. 4*D*). This result suggests that alisertib treatment caused a change in either Puma or Mcl-1, which resulted in an enhanced binding between these two proteins. As the binding of Mcl-1 to Noxa and BimEL did not seem to change, it is likely that a change in Puma’s intrinsic property may have caused the enhanced binding, and such binding contributes to the inactivation of Mcl-1 in an alisertib-induced fashion. Although truncated Bid (tBid) was not detected after alisertib treatment, it remains possible that a fraction of Bid is cleaved during the treatment.

### Cleavage of Bid is required for alisertib–ABT-737-induced apoptosis

**Figure 5:**
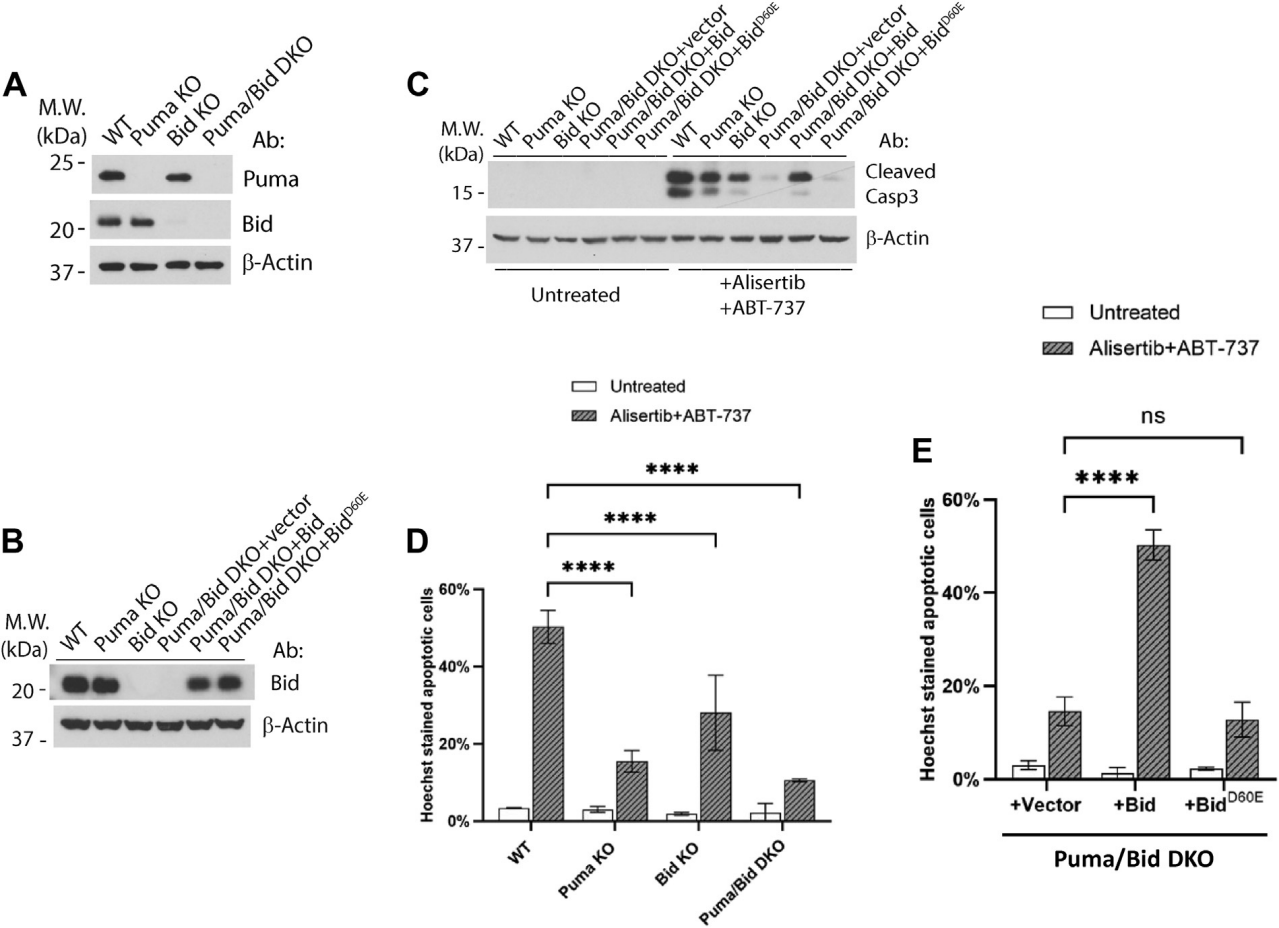
**Cleavage of Bid is required for Alisertib–ABT-737-induced apoptosis.***A*, Puma KO, Bid KO, and Puma–Bid DKO were generated from HCT116 cells by CRISPR–Cas9, the whole-cell lysate was used for Western blot to examine KO. *B*, expression of Bid and Bid^D60E^ mutant in Puma–Bid DKO cells was by retroviral infection. Cells were harvested, and the whole-cell lysate was used to examine protein expression *via* Western blot. *C*, cells established in (*A*) and (*B*) were treated with alisertib (1 μM) for 24 h with ABT-737 (1 μM) for 6 h, cells were harvested, and the whole-cell lysate was used for Western blot analysis. *D*, KO cell lines established in (*A*) were treated with alisertib (1 μM) for 24 h with ABT-737 (1 μM) for 6 h. Cells were subjected to Hoechst staining for quantification of apoptotic cells. Mean and SD values were shown on the graph, n = 3. *E*, rescue cell lines established in (*B*) were treated with alisertib (1 μM) for 24 h with ABT-737 (1 μM) for 6 h. Cells were subjected to Hoechst staining for quantification of apoptotic cells. Mean and SD values were shown on the graph, n = 3. Representatives of at least three independent experiments were shown for Western blots. Two-way ANOVA was used to test the significance, *p* < 0.0001 was defined as significant and indicated with ∗∗∗∗, ns represented not significant.

Since Bid is clearly playing a role in alisertib–ABT-737-induced apoptosis (Fig. 4, *A*–*C*), we investigated whether cleavage of Bid is necessary in this pathway. As binding of tBid to Mcl-1 following alisertib treatment is difficult to detect by Western blot, we resorted to a functional strategy to test whether cleavage of Bid is required for apoptosis following alisertib–ABT-737 treatment. First, we used CRISPR–Cas9 to generate Puma–Bid DKO HCT116 cells (Fig. 5*A*). While Bid KO and Puma KO cells each reduced apoptosis following alisertib–ABT-737 treatment by approximately 50%, the Puma–Bid DKO cells showed a stronger block (Fig. 5, *C* and *D*), indicating that Bid and Puma are in two parallel pathways, and both contributed to killing by alisertib–ABT-737 treatment. Second, the Puma–Bid DKO cells should allow us to examine the requirement of Bid cleavage in this apoptotic pathway through reconstitution. We therefore used retrovirus to express either wildtype Bid or its D60E mutant, which is known to be refractory to caspase-2/-3/-8-mediated cleavage (36, 37, 38) (Fig. 5*B*). As expected, while expression of the wildtype Bid restored apoptosis to the level of apoptosis in Puma KO cells, the expression of Bid^D60E^ mutant failed to restore apoptosis (Fig. 5, *C* and *D*), indicating that cleavage of Bid, presumably by a caspase, is required for alisertib–ABT-737-induced apoptosis.

### Activation of caspase-2 is required for alisertib–ABT-737-induced apoptosis

**Figure 6:**
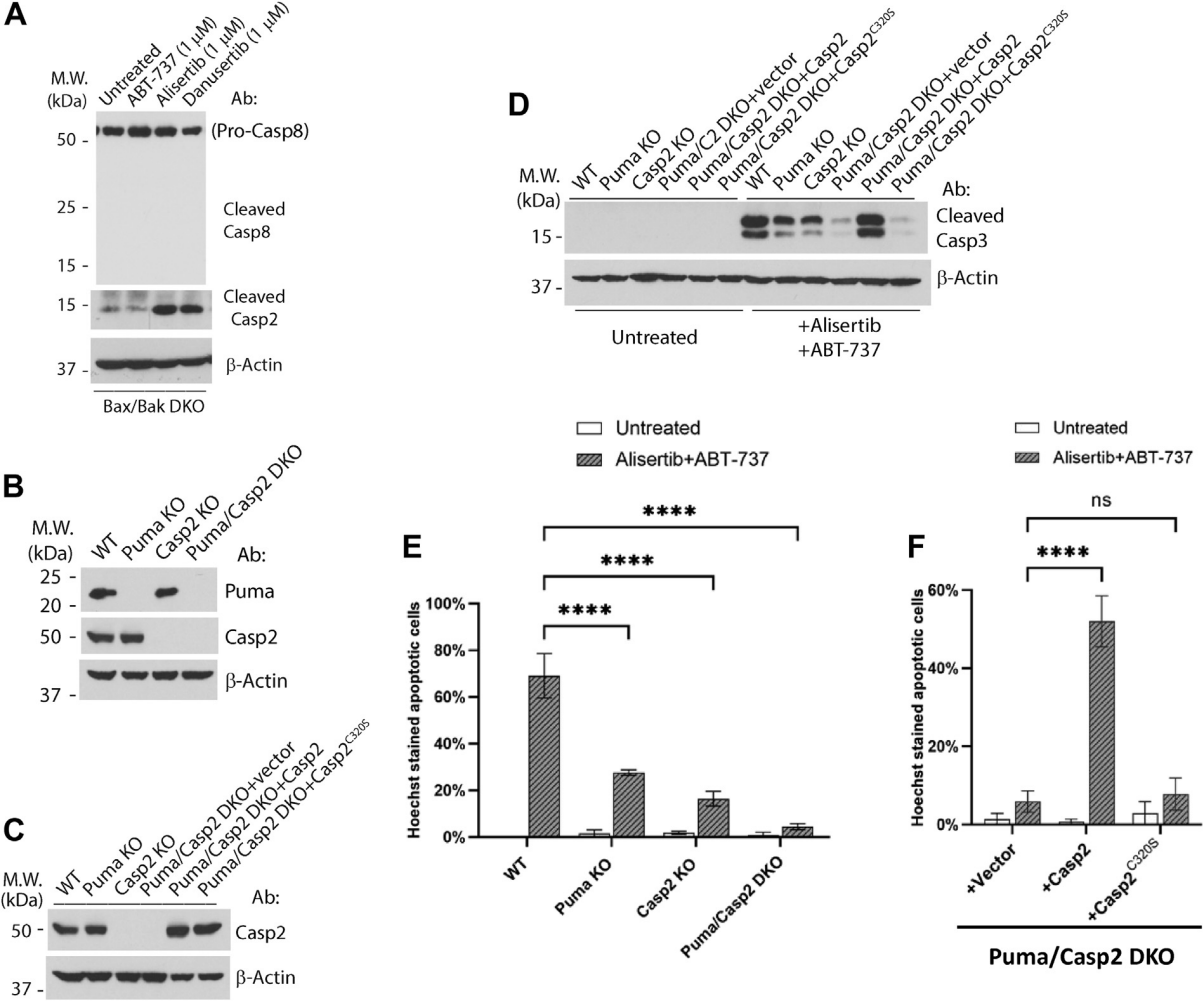
**Activation of caspase-2 is required for alisertib–ABT-737-induced apoptosis.***A*, Bax/Bak double KO (DKO) cells were treated with ABT-737, alisertib, and danusertib at the indicated concentrations for 24 h and were harvested for Western blot analysis. *B*, Puma KO, Caspase-2 KO (Casp2 KO), and Puma/Caspase-2 DKO (Puma/Casp2 DKO) were generated from HCT116 cells by CRISPR–Cas9, and the whole-cell lysate was used to examine KO by Western blot. *C*, expression of Caspase-2 (Casp2) and Caspase-2^C320S^ (Casp2^C320S^) mutant in Puma/Casp2 DKO was by retroviral infection. Cells were harvested, and the whole-cell lysate was used to examine protein expression *via* Western blot. *D*, cells established in (*B*) and (*C*) were treated with alisertib (1 μM) for 24 h with ABT-737 (1 μM) for 6 h, cells were harvested, and the whole-cell lysate was used for Western blot analysis. *E*, KO cell lines established in (*B*) were treated with alisertib (1 μM) for 24 h with ABT-737 (1 μM) for 6 h. Cells were subjected to Hoechst staining for quantification of apoptotic cells. Mean and SD values were shown on the graph, n = 3. *F*, rescue cell lines established in (*C*) were treated with alisertib (1 μM) for 24 h with ABT-737 (1 μM) for 6 h. Cells were subjected to Hoechst staining for quantification of apoptotic cells. Mean and SD values were shown on the graph, n = 3. Representatives of at least three independent experiments were shown for Western blots. Two-way ANOVA was used to test the significance, *p* < 0.0001 was defined as significant and indicated with ∗∗∗∗, ns represented not significant.

Bid is known to be cleaved by caspase-8, 3/7, and 2 (36, 38, 39, 40). We therefore examined the activation/cleavage of either caspase-8 or caspase-2 following ABT-737, alisertib, or danusertib. As shown in Figure 6*A*, while caspase-8 was not cleaved, caspase-2 was clearly cleaved following either alisertib or danusertib treatment. We therefore examined the requirement of caspase-2 in alisertib–ABT-737-induced apoptosis in HCT116 cells through CRISPR–Cas9. Caspase-2 KO cells displayed a 40 to 50% decrease in apoptosis, indicating that caspase-2 is involved in this pathway. If caspase-2 is responsible for the cleavage of Bid, we would expect that cells deficient for both caspase-2 and Puma display similar apoptotic response to those deficient for both Bid and Puma. We therefore generated caspase-2/Puma DKO HCT116 cells (Fig. 6*B*). Indeed, the caspase-2/Puma DKO cells showed a much stronger blockade in apoptosis than caspase-2 KO or Puma KO. To validate the phenotype of the caspase-2/Puma DKO, and test the requirement of caspase-2 activity, we used the retrovirus to express either wildtype caspase-2 or its catalytically dead mutant, Casp2^C320S^, in the DKO cells (Fig. 6*C*). Apoptosis in these DKO cells was restored to the level of Puma KO cells by expressing wildtype caspase-2, but not Casp2^C320S^, indicating that active caspase-2 is critically involved in alisertib–ABT-737-induced apoptosis (Fig. 6, *D* and *E*). As caspase-2 is known to cleave Bid at D60 *in vitro* (40), and that cleavage of Bid at D60 is required for the apoptotic activity of Bid in the cells (37), these results strongly suggest that alisertib–ABT-737-induced apoptosis requires the caspase-2–Bid axis.

## Discussion

**Figure 7:**
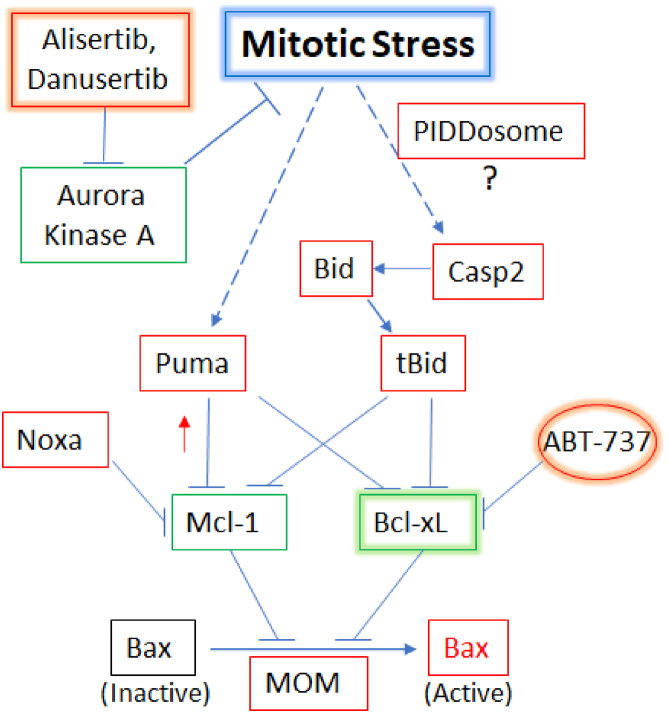
**Model for mitotic stress-induced apoptosis.** Diagram of the putative pathway of mitotic catastrophe–induced apoptosis based on the current study. MOM, mitochondrial outer membrane.

Inhibitors of AURKs are known to induce mitotic catastrophe and are considered promising candidate antitumor drugs (1). However, their abilities to induce cell death have not been well documented. In this study, we found that alisertib and danusertib are inefficient inducers of apoptosis in colon cancer cells, and yet, they potently induce apoptosis when combined with the BH3-mimetic ABT-737. We identified the Bcl-2 family proteins, Bcl-xL, Bax, Puma, Bid, Noxa, as critical players in this apoptosis pathway (Fig. 7).

### Caspase-2–Bid axis as a major determinant for AURK inhibitor–induced apoptosis

Both caspase-2 and Bid have been shown to be involved in mitotic stress–induced apoptosis (39, 40, 41). It is therefore not surprising that they are essential for alisertib–ABT-737-induced apoptosis. However, our results in colon cancer cells for the first time establish activation of caspase-2 and cleavage of Bid as two key events during AURK inhibitor–ABT-737-induced apoptosis. As alisertib treatment alone is sufficient to activate caspase-2 without activating caspase-8 or caspase-3, which have also been known to cleave Bid, it strongly suggests that Bid is cleaved by caspase-2 but not by caspase-8 or caspase-3 during alisertib treatment. Since caspase-2 efficiently cleaves Bid *in vitro*, and such cleavage is sufficient to induce cytochrome *c* release (39, 40), it is likely that Bid is cleaved by caspase-2 to generate tBid for the activation of Bax. It is also important to point out that our results for the first time demonstrated that the caspase-2–Bid axis following AURK inhibition is insufficient to induce apoptosis in certain colon cancer cell lines. Although tBid is generated and able to bind both Mcl-1 and Bcl-xL (24), the amount of tBid generated by caspase-2 is apparently not sufficient to inactivate endogenous Bcl-xL in these cells, as evidenced by the requirement for ABT-737 in the induction of apoptosis. In addition, the mechanism of caspase-2 activation during mitotic catastrophe has not been fully elucidated (42). Although the PIDDosome is a likely candidate that activates caspase-2 after alisertib treatment, yet PIDDosome-independent activation of caspase-2 has also been reported (41).

### How is Mcl-1 suppressed by alisertib treatment?

Noxa is a transcriptional target of p53, and it exerts its apoptotic function primarily by binding to and inactivating Mcl-1 (24, 43). Although Noxa’s protein level does not increase, its loss is sufficient to block alisertib–ABT-737-induced apoptosis (Fig. 4). These observations support the notion that under normal circumstances, Noxa is constitutively in complex with Mcl-1, effectively limiting the basal level of active Mcl-1 in the cell (33, 34). Elimination of Noxa is therefore liberating Mcl-1, which suppresses Bax and Bak, blocking their activation. On the other hand, alisertib treatment significantly enhanced the binding of Puma to Mcl-1 without a noticeable increase of Puma’s protein level (Fig. 4). The mechanism of such increase, however, is unclear. Puma is a transcriptional target of the tumor suppressor p53 and yet can be upregulated through both p53-dependent and p53-independent mechanisms in response to various stress signals (44, 45, 46). In addition, phosphorylation of Puma has been shown to stabilize Puma (47). However, the lack of an obvious increase of Puma in protein level does not support the involvement of a transcriptional upregulation or a phosphorylation-mediated stabilization of Puma following the alisertib treatment. We speculate that a yet unidentified post-translational modification on either Puma or Mcl-1 is responsible for the enhanced binding. Alternatively, it is possible that an unidentified inhibitor of Puma–Mcl-1 interaction is lost during alisertib treatment. Although it is currently unclear whether this induced binding to Mcl-1 is sufficient for Bax activation upon mitotic stress, our data (Fig. 4) suggest that this binding contributes to inactivation of Mcl-1 and the subsequent Bax activation. Of note, tumor suppressor p53 is upregulated during alisertib treatment (Fig. S3) in a Bax/Bak-independent fashion; it is therefore of importance to examine the involvement of p53 in the activation of both Puma and Bid during AURK inhibition in the future.

### ABT-737–Alisertib as a combinatorial therapy against cancer?

Alisertib is mostly known to induce senescence in cancer cells (10). The findings that Bcl-xL is the major suppressor of apoptosis following treatment by alisertib and that ABT-737 cooperates with alisertib in apoptosis induction in colon cancer cells raise the possibility that senescent cancer cells are mainly kept alive by Bcl-xL. In other words, Bcl-xL may be essential for the manifestation of the senescence phenotype in at least some cancer cells. It is possible that in colon cancer cells, the expression of Bcl-xL is higher than some other cancer cells. Alternatively, the antiapoptotic activity of Bcl-xL is enhanced through unknown mechanisms following AURK inhibitor treatment. Indeed, colon cancer cell lines, especially HCT116 and DLD-1, are among the various cancer cell lines that have higher expression levels of Bcl-xL (Fig. S4*A*). However, the expression of Bcl-xL in colon tumor samples is moderate (Fig. S4*B*) and is not significantly associated with prognosis among colon cancer patients according to The Cancer Genome Atlas dataset. Regardless, the prominent role of Bcl-xL in the survival of senescent cells is consistent with the finding that navitoclax, a slightly modified and orally available version of ABT-737, is generally considered a senolytic drug (48, 49). *In vivo* mouse studies and possibly human patient trials are necessary to investigate whether navitoclax is able to cooperate with alisertib for the killing of hematological and solid tumors. Future mouse xenograft studies are highly desirable to examine the potential of AURK and Bcl-xL as drug targets in cancer. Of importance, alisertib has been shown to be safe for human (14), and although navitoclax has been found to cause thrombocytopenia, this may be manageable to certain extent through changes in dosing and pharmacological intervention (50).

In summary, this study defined the Bcl-2 network required for AURK inhibition–induced apoptosis and suggested that a combinatorial inhibition of AURKs and Bcl-xL may be a potent inducer of apoptosis in colon cancer cells (Fig. 7). It will be of great interest to examine the therapeutic efficacy of such combinatorial therapy in patients with solid tumor.

## Experimental procedures

### Cell culture

HCT116 cells (American Type Culture Collection) were cultured in McCoy’s 5A medium, and DLD-1 cells (a gift from Dr Jennifer Black’s laboratory) were cultured in RPMI1640 medium with 100 IU/ml penicillin/100 mg/ml streptomycin and 10% fetal bovine serum. 293GP cells were cultured in Dulbecco's modified Eagle's medium with the same concentration of penicillin/streptomycin and fetal bovine serum as mentioned previously. All cells were maintained at 37 °C with 5% CO_2_.

### Reagents

Alisertib (MLN8237) (catalog no.: S1133), danusertib (PHA-739358) (catalog no.: S1107), doxorubicin (adriamycin) HCl (catalog no.: S1208), and bortezomib (PS-341) (catalog no.: S1013) were purchased from SelleckChem and dissolved in dimethyl sulfoxide (DMSO). ABT-737 (catalog no.: 852808-04-9) and S63845 (catalog no.: 1799633-27-4) were purchased from ChemieTek and dissolved in DMSO. Camptothecin (catalog no.: 159732) and z-VAD-FMK (catalog no.: 03FK1090-CF) were purchased from MP Biomedicals and dissolved in DMSO. Human recombinant TRAIL was generated as previously described (51). 1,6-Bismaleimidohexane was purchased from Thermo Fisher Scientific (catalog no.: 22330). Annexin V-FITC was purchased from BioLegend (catalog no.: 640906). Propidium iodide (PI) solution was purchased from G-Biosciences (catalog no.: 786-1272).

Antibodies used for immunoblotting include anti-β-actin (Sigma–Aldrich; catalog no.: A5441), anti–caspase-3 (Santa Cruz Biotechnology; catalog no.: sc-56053), anti-GFP (Santa Cruz Biotechnology; catalog no.: sc-9996), anti-MCL-1 (Santa Cruz Biotechnology; catalog no.: sc-819), anti-BCL-2 (Cell Signaling Technology; catalog no.: 15071), anti-Bcl-xL (Cell Signaling Technology; catalog no.: 2762), anti-BCL-w (Cell Signaling Technology; catalog no.: 2724), anti-Bax (Cell Signaling Technology; catalog no.: 2772), anti-Bak (Cell Signaling Technology: catalog no.: 5023), anti-PUMA (Cell Signaling Technology; catalog no.: 12450), anti-Bid (36), anti-Bim-EL (Cell Signaling Technology; catalog no.: 2819), anti-BAD (Santa Cruz Biotechnology; catalog no.: sc-3044), anti-Noxa (Santa Cruz Biotechnology; catalog no.: sc-515840), anti-BIK (Cell Signaling Technology; catalog no.: 4592), anti–caspase-2 (Cell Signaling Technology; catalog no.: 2224), anti–caspase-8 (Cell Signaling Technology; catalog no.: 9746S), anti-p53 (Santa Cruz Biotechnology; catalog no.: sc-393), and anti-GFP (Santa Cruz Biotechnology; catalog no.: sc-459).

Secondary antibodies include anti-rabbit (Sigma–Aldrich; catalog no.: A6154) and antimouse (Jackson ImmunoResearch, Inc; catalog no.: 715-035-150).

### Plasmids

CRISPR and TALEN plasmids used were reported in an earlier study (23). The sgRNA sequence of CASPASE-2 cloned into px330 is TGGTGAGCAACATATCCTCC.

The retroviral plasmids pMaRX-9His-GFP and pMaRX-9His-MCL-1 were described before (34).

The retroviral expression plasmids pMSCV PIG (Puro IRES GFP empty vector) (plasmid #21654) was purchased from Addgene. The pMSCV PIG-BID and PIG-BID-D60E were cloned into pMSCV PIG by restriction site XhoI/EcoRI. pcDNA3-Casp2-FLAG (plasmid #11811) was purchased from Addgene. The retroviral expression plasmids mpMIG-caspase-2 and mPMIG-caspase-2^C320S^ were cloned into XhoI/EcoRI-digested mpMIG.

### Western blot

Cells were harvested and lysed in EBC buffer (50 mM Tris–HCl, 120 mM NaCl, 0.5% v/v NP-40, pH 8.0) with protease inhibitors and PMSF by rotating in 4 °C for 2 h. The supernatant was collected as the whole protein lysate after being centrifuged at 22,000*g* for 10 min at 4 °C. The concentration of protein was measured by Coomassie Protein Assay (Thermo Fisher Scientific; catalog no.: 1856209). After mixing with 4× SDS loading buffer, the whole protein lysate was boiled at 100 °C for 5 min. Protein samples were loaded into 8% or 12% SDS-PAGE gels and ran for 45 to 50 min at constant 200 voltage and transferred onto a nitrocellulose membrane at constant 400 mA for 1 h. The membrane was incubated with primary antibody for Western blotting overnight at 4 °C, after which it was washed by PBS with Tween-20 for three times, then incubated with secondary antibody at room temperature for 2 h. Before developing films, the membrane was washed by PBS with Tween-20 for three times.

### Virus production and infection

The transfection of retroviral production of retroviruses was performed in 293GP cells. 239GP cells were seeded in 60 mm plates the day before transfection at 50% confluency. About 2 μg target plasmid with 0.5 μg envelope plasmid VSV.G (Addgene #14888) and transfection agent polyethyleneimine (MilliporeSigma; catalog no.: 408727) were mixed in pure Dulbecco's modified Eagle's medium and transfected into 293GP cells. Viruses were harvested 48 h post-transfection. Virus infection was performed by 10 μg/ml polybrene (Santa Cruz; catalog no.: NC9840454) for 24 h. Cells infected mPMIG viruses were sorted by flow cytometry for GFP positive. Cells infected with pMaRX and PIG viruses were selected by puromycin at 1 μg/ml.

### Nickle beads pull-down

Treated cells were harvested and washed three times with PBS. Cells were lysed in EBC buffer with 1 mM PMSF and protease inhibitor followed by rotation at 4 °C for 1 h. Nickel–nitrilotriacetic acid (Ni–NTA) agarose (Qiagen) was washed three times and resuspended with EBC buffer before being mixed with the protein lysate. The lysate was centrifuged at 22,000*g* for 10 min, and the supernatant was incubated with 100 μl slurry of Ni–NTA agarose. After 2 h of rotation at 4 °C, the beads were washed three times by EBC buffer containing 10 mM of imidazole. SDS loading dye and EBC buffer with 250 mM imidazole was used to elute proteins from Ni–NTA beads.

### CRISPR–Cas9 transfection

Cells were seeded in 35 mm plates at 50% confluency 24 h before transfections. About 500 ng CRISPR plasmid was cotransfected with 500 ng of the corresponding mRFP-TS-2A-HYG-EGFP reporter plasmid. pcDNA3.1 was added to make the total DNA concentration of 2 μg per transfection. Medium was changed 24 h after transfection. About 48 h after transfection, transfected cells were subjected to flow cytometry sorting for GFP-positive cells. The sorted cells were seeded in 15 cm plates for single clones.

### Hoechst staining

Cells were seeded in 6-well plates 24 h before treatments. After treatments, cells were stained with 1 μg/ml Hoechst 33342 (Molecular Probes; catalog no.: H3570) for 30 min in cell culture incubator. Pictures were taken for two to three random viewing areas containing around 1000 cells for each plate. Pictures were cropped randomly, and each cropped ones contained around 100 to 200 cells. For each group, four cropped pictures were randomly selected and counted. Cells undergoing nuclear condensation were considered apoptotic cells. Mean value of the apoptotic percentage of all counted pictures was compared.

### Annexin V–PI staining

Cells were stained with FITC Annexin V and PI according to the manufacturer’s instructions. About 10,000 cells from each sample were analyzed by flow cytometry. Cell counting and analysis were performed on a BD FACSCalibur flow cytometer with BD FACSDiva 8.0 software (Flow Cytometry Research Facility, University of Nebraska Medical Center).

### Statistical analysis

GraphPad Prism 6.0 (GraphPad Software) was used for statistical analysis. Data are shown as the mean ± SD. Two-way ANOVA was used for comparing variables of independent groups, *p* < 0.0001 was considered statistically significant.

## Data availability

All representative data are contained within the article.

## Supporting information

This article contains supporting information.

## Conflict of interest

The authors declare that they have no conflicts of interest with the contents of this article.
