# Supplementary material for: Combined inhibition of aurora kinases and Bcl-xL induces apoptosis through select BH3-only proteins
Source: J Biol Chem. 2023 Jan 6;299(2):102875. doi: 10.1016/j.jbc.2023.102875 (PMC9922828; doi:10.1016/j.jbc.2023.102875)
Supplement: Supplemental Figures S1–S4 [file mmc1.pdf]

SUPPLEMENTARY FIGURE LEGENDS

Supplementary Figure S1. ABT-737 sensitizes HCT116 and DLD-1 cells to Alisertib and Danusertib-induced apoptosis. (A) HCT116 WT cells were treated with Alisertib (1 μM) for 24 hours with or without ABT-737 (1 μM) for 6 hours. Cells were harvested and stained by Annexin V-FITC and PI followed by flow cytometry analysis. Mean and SD values of early and late apoptotic cells were shown in the graph, n=3. **(B)** Mean and SD values of total apoptotic cells from (A) were shown in the graph, n=3, One-way ANOVA was used to test the significance, $P<0.0001$ was defined as significant and indicated with ****. **(C)** Same treatments and assay as described in (A) were done in DLD-1 WT cells. Mean and SD values of early and late apoptotic cells were shown in the graph, n=3. **(D)** Mean and SD values of total apoptotic cells from (C) were shown in the graph, n=3, One-way ANOVA was used to test the significance, $P<0.0001$ was defined as significant and indicated with ****.

Supplementary Figure S2. Bcl-xL is endogenously expressed at a higher level than Bcl-2 in HCT116 cells. (A) Expression of GFP, GFP-Bcl-xL and GFP-Bcl-2 in Bcl-xL KO cells was done by retroviral infection. Cells were sorted by low (L) and high (H) GFP intensity via flow cytometry. Sorted cells were harvested and subjected to western blot. **(B)** Cell lysates were diluted as indicated and loaded on SDS-PAGE for western blot with the indicated antibodies. **(C)** Sorted cells from (A) were treated with Alisertib (1 μM) for 24 hours. Cells were harvested and the whole cell lysate was for western blot analysis.

Supplementary Figure S3. p53 is up regulated in response to Alisertib and Danusertib. WT and Bax/Bak DKO HCT116 cells were treated with Alisertib (1 μM) and Danusertib (1 μM) and cells were harvested at indicated time points. The whole cell lysate was used for western blot analysis.

Supplementary Figure S4. Bcl-xL expression in cancer cell lines, tumors, and its relationship with prognosis of colorectal cancer patients. (A) Bcl-xL expression of various cell lines are acquired through Cancer Cell Line Encyclopedia (CCLE) database based on DepMap Public 22Q2 database the DepMap Portal. The box plot of colon/colorectal cancer are labeled in red. HCT116 and DLD-1 cells are indicated as

green dots. **(B)** BclxL expression of patient samples are accessed through TCGA Pan Cancer (PANCAN) dataset with Xena Browser, along with primary disease type of each sample. The exported data is grouped according to primary disease type, and colon cancer is shown in red in the graph.

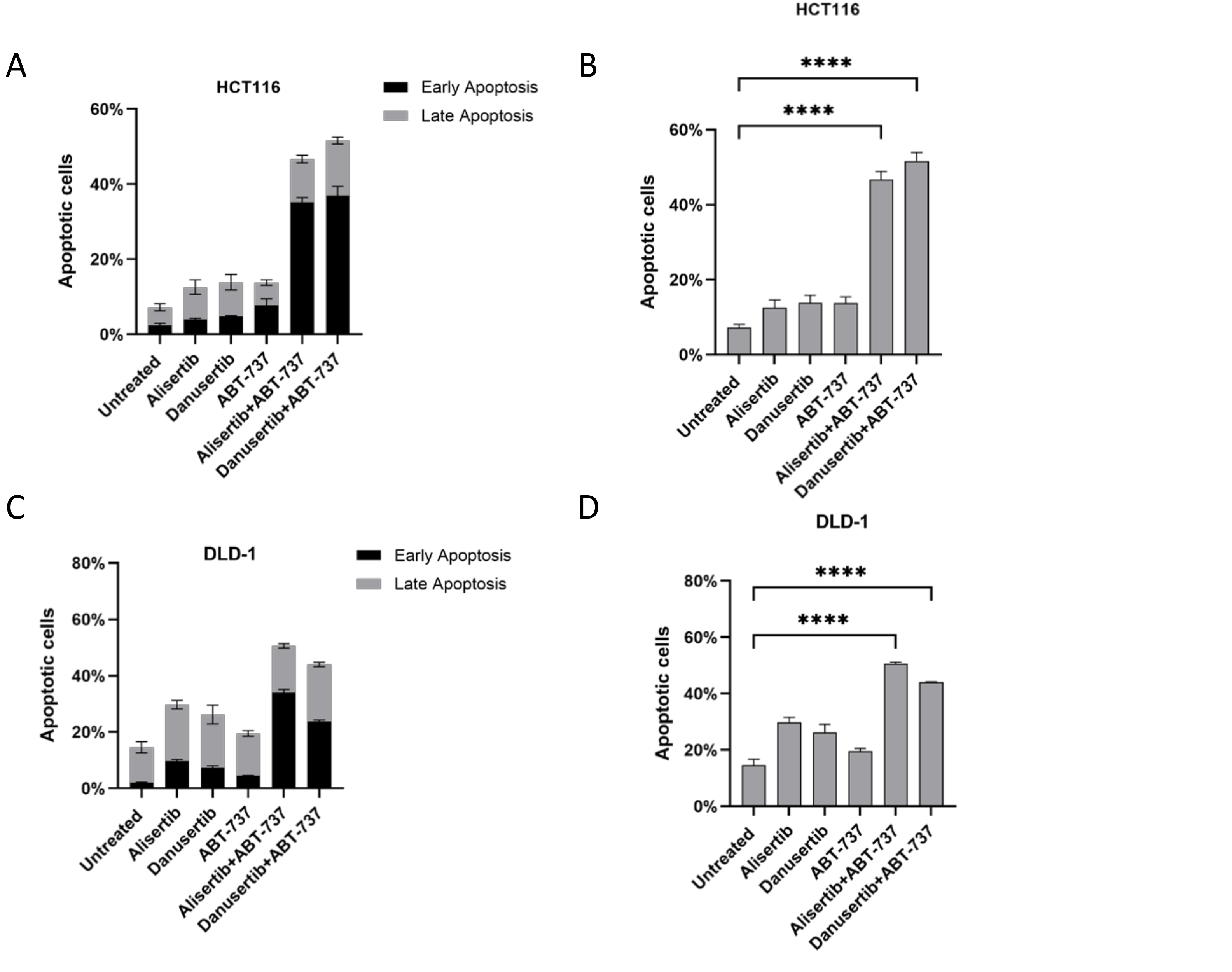

Supplementary Fig. S1. ABT-737 sensitize HCT116 and DLD-1 cells to Alisertib and Danusertib-induced apoptosis.

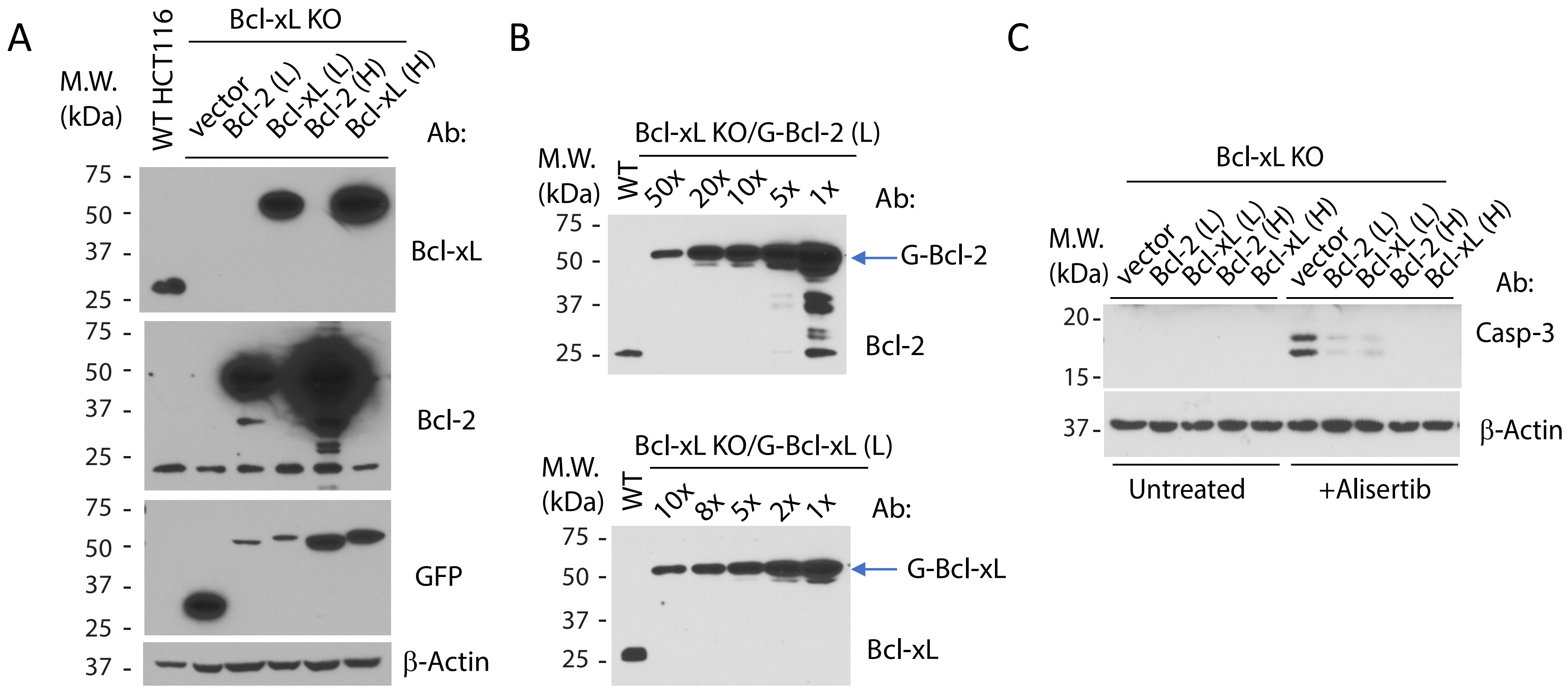

Supplementary Fig. S2. Bcl-xL is endogenously expressed at a higher level than Bcl-2 in HCT116 cells.

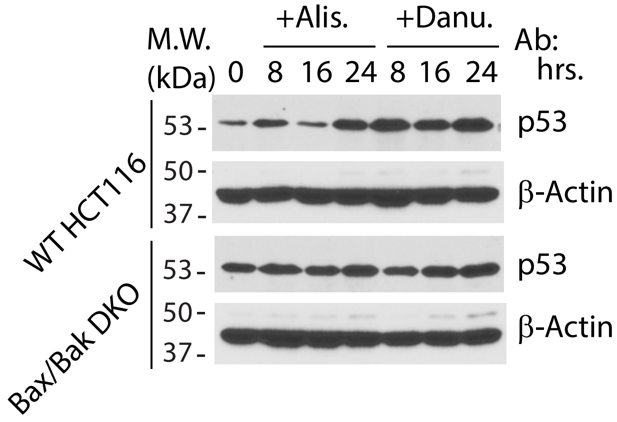

Supplementary Fig. S3. p53 protein is up-regulated in response to Aliertib and Danusertib.

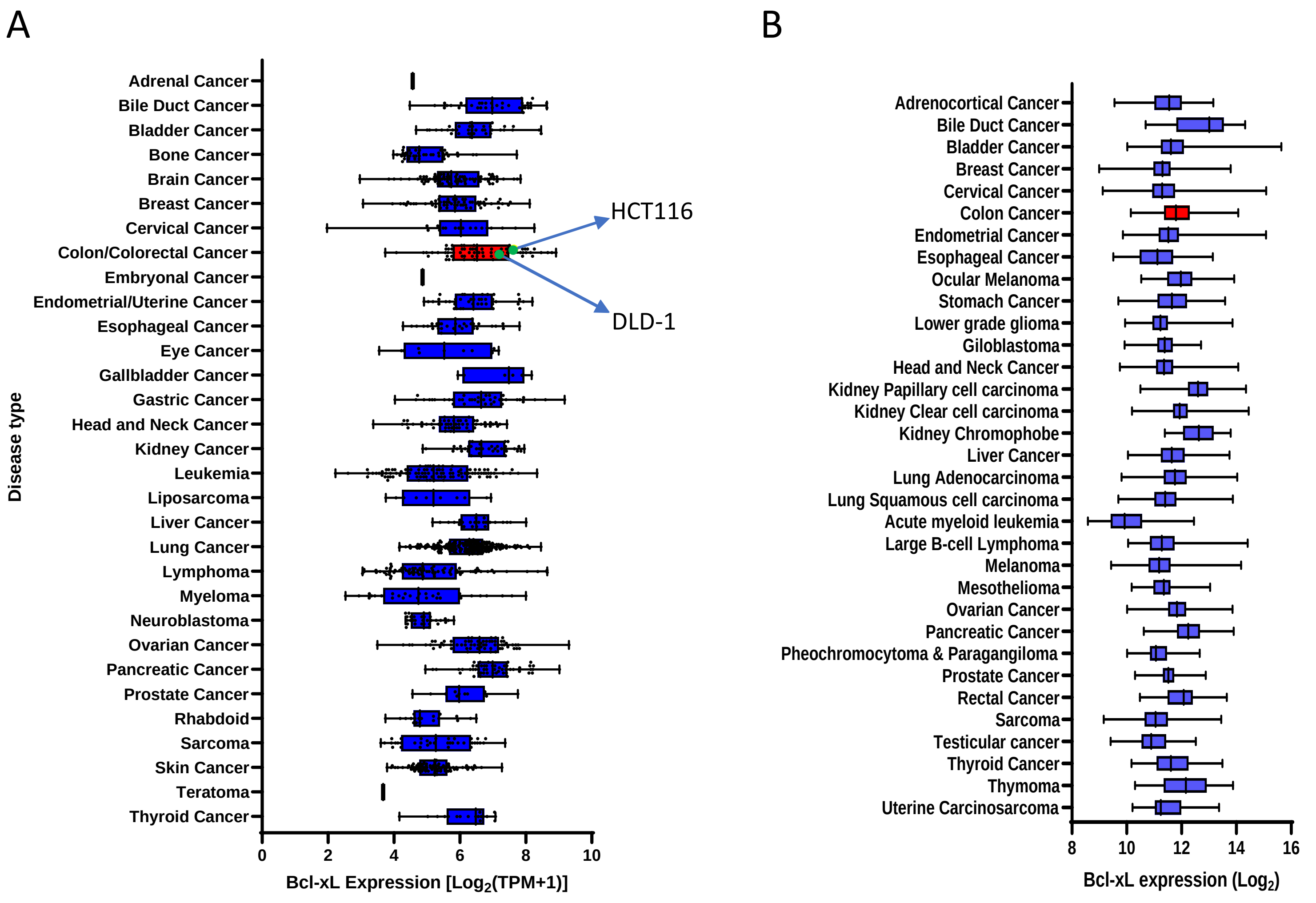

Supplementary Fig. S4. Bcl-xL expression in cancer cell lines and tumors.
